# Cochlear Implantation in Charcot-Marie-Tooth Disease: Case Report and Review of the Literature

**DOI:** 10.1155/2018/1760978

**Published:** 2018-03-26

**Authors:** C. Lane Anzalone, Sarah Nuhanovic, Amy P. Olund, Matthew L. Carlson

**Affiliations:** ^1^Department of Otorhinolaryngology, Mayo Clinic, Rochester, MN, USA; ^2^Department of Laboratory and Research, Mayo Clinic, Rochester, MN, USA; ^3^Department of Audiology, Mayo Clinic, Rochester, MN, USA

## Abstract

**Introduction:**

Charcot-Marie-Tooth (CMT) disease is a peripheral hereditary neuropathy associated with motor and sensory impairment and can result in profound sensorineural hearing loss (SNHL). Currently, the role of cochlear implantation in the setting of CMT and other progressive peripheral neurodegenerative disorders is not well established.

**Methods:**

Case report and review of the English literature.

**Results:**

A 70-year-old male with CMT was referred for evaluation of progressive asymmetric SNHL and reported a 15-year duration of deafness involving the left ear. Audiometric testing confirmed profound SNHL in the left ear, while the right ear exhibited moderate-to-severe SNHL. Left-sided cochlear implantation was performed using a conventional length lateral wall electrode. Intraoperative device testing found normal impedance levels throughout the array; however, electrically evoked auditory potentials were absent on all electrodes. Upon initial activation 3 weeks after surgery, the patient reported excellent access to sound in the cochlear implant-only condition. He has made good progress at each subsequent visit; speech perception testing after seven months showed improvement from 0% to 32% on AzBio sentence and 53% on CNC phoneme testing in the cochlear implant-only condition.

**Conclusion:**

We report the third case of cochlear implantation in a patient with CMT. SNHL in CMT is hypothesized to result from disruption of synchronous activity of the cochlear nerve. In patients with CMT, cochlear implantation may reconstitute synchronous neural activity by way of supraphysiological electrical stimulation. Our results corroborate two earlier reports that cochlear implantation is a viable option for rehabilitation of SNHL in this unique subset of patients.

## 1. Introduction

Charcot-Marie-Tooth (CMT) disease is a clinically and genetically heterogeneous group of disorders affecting the peripheral nervous system. The disease can manifest in profound SNHL, hypothesized to result from disruption of synchronous activity of the cochlear nerve. Given that CMT affects the retrocochlear auditory pathway, there have been theoretical concerns that CI is incapable of providing meaningful auditory stimulation. Two previous cases of CI in patients with CMT have been described in the world literature; thus, the role of cochlear implants in auditory rehabilitation of CMT-associated SNHL is poorly defined [1, 2]. We present a patient with CMT-associated deafness successfully treated with cochlear implantation.

## 2. Case Presentation

A 70-year-old male with CMT was referred for evaluation of progressive asymmetric SNHL and reported a 15-year duration of deafness involving the left ear. He was initially a bilateral hearing aid user but subsequently stopped using his hearing aid in the left ear several years prior due to experiencing progressive audiometric decline. Audiometric testing confirmed profound SNHL in the left ear, while the right ear exhibited moderate-to-severe SNHL. Information regarding the subtype of his CMT is unavailable at our tertiary care facility; prior workup was performed at another institution.

Left-sided cochlear implantation was performed using a conventional length lateral wall electrode. The electrodes were introduced without resistance, and a full insertion was achieved using the MED-EL™ Synchrony Flex® 28 cochlear implant. Intraoperative device testing found normal impedance levels throughout the array; however, electrically evoked auditory potentials were absent on all electrodes. Upon initial activation 3 weeks after surgery, the patient reported excellent access to sound in the cochlear implant-only condition. He has made good progress at each subsequent visit; speech perception testing after seven months showed improvement from 0% preoperatively to 32% on AzBio sentence and 53% on CNC phoneme testing in the cochlear implant-only condition. At an 18-month phone follow-up, he reports improving subjective benefit and consistent usage of the device.

## 3. Discussion

Charcot-Marie-Tooth disease is the most commonly occurring hereditary motor and sensory neuropathy. CMT can be classified as types 1 to 7, X, and intermediate, with many subtypes. CMT is genetically heterogeneous with more than 30 genes identified to date. Despite this, the vast majority of cases are attributed to mutations in just four of these genes: *PMP22*, *MPZ*, *GJB1*, and *MFN2*. CMT types 1 and 2 represent by far the largest proportion of patients; these classifications are based off features of demyelinating (CMT1) or axonal (CMT2) dysfunction. Motor weakness is the predominant symptom, typically arising in the first or second decade of life. The majority of treatment options are directed towards alleviation of motor deterioration; there have been few reports describing the management of hearing loss in patients with CMT [3].

Hearing loss in patients with CMT is associated with point mutations or deletions in the peripheral myelin protein (PMP) 22 gene, a myelin regulatory gene [4]. Neural conduction abnormalities in the auditory pathway lead to the auditory deficits seen in individuals with CMT. While the exact pathogenesis of deafness in CMT patients is uncertain, it has been proposed that the resultant SNHL is a consequence of retrocochlear dysfunction. The failure to detect auditory brainstem responses is theorized to result from the loss of discharge synchrony secondary to demyelination or axonal disruption of the cochlear nerve [5, 6].

Traditionally, retrocochlear disease affecting the auditory pathway has been considered a contraindication to CI. In patients with auditory neuropathy spectrum disorder (ANSD), there is an absence of auditory brainstem responses despite the intact outer hair cell function. ANSD results in poor speech discrimination while general awareness of speech is often retained. The resultant severe impairment in temporal processing ability was originally thought to be unchanged by CI; however, over the last decade, we have seen several disease processes that have challenged this notion. Specifically, it is well documented that many patients with auditory nerve hypoplasia, auditory neuropathy, and acoustic neuroma with an intact cochlear nerve gain significant benefit from CI [7, 8]. In patients with CMT, cochlear implantation may reconstitute synchronous neural activity by way of supraphysiological electrical stimulation, thus improving speech discrimination scores.

Two prior cases of cochlear implantation in patients with SNHL attributed to CMT have been reported in the world literature [1, 2]. Each demonstrated improvements in speech discrimination scores. The improvement progression was noted to be slower than in the typical subset of patients receiving CI; however, significant benefit was eventually realized. Goswamy et al. described a 67-year-old male with CMT-attributed SNHL who underwent CI. Open-set speech discrimination (BKB sentences) scores were obtained at 1 week, 3 months, 9 months, and 21 months after activation and were noted to be 0, 0, 53, and 54%, respectively. Postelmans et al. described a 53-year-old female with CMT who first experienced a progressive hearing loss at 8 years of age; she had worn hearing aids for 25 years prior to undergoing CI. At 6 months postoperatively, she had improved to 59% word discrimination at 60 dB from preoperative discrimination of 50% at 75 dB. In our patient, similar findings were observed. We attribute his initial absent evoked auditory potentials to his extent and duration of deafness. A gradual improvement has been noted with each subsequent visit, and the patient has been pleased with his progress to date. It is important to note that his duration of deafness (15 years) should also be considered when evaluating his rate of progress.

## 4. Conclusion

We report the third case of cochlear implantation in a patient with CMT. Improvement of speech discrimination in this unique subset of patients has been shown to occur at a more gradual rate of progression. While no definitive recommendations concerning the efficacy of CI in patients with CMT have been established, our results corroborate two earlier reports that cochlear implantation is a viable option for rehabilitation of SNHL in CMT patients. Future study should be performed in an attempt to further define the role of CI in the setting of CMT.
